# Patient safety culture at neonatal intensive care units: perspectives of the nursing and medical team^1^

**DOI:** 10.1590/0104-1169.3624.2477

**Published:** 2014

**Authors:** Andréia Tomazoni, Patrícia Kuerten Rocha, Sabrina de Souza, Jane Cristina Anders, Hamilton Filipe Correia de Malfussi

**Affiliations:** 2 Master's student, Universidade Federal de Santa Catarina, Florianópolis, SC, Brazil. Scholarship holder from Coordenação de Aperfeiçoamento de Pessoal de Nível Superior (CAPES), Brazil. RN, Hospital Universitário Polydoro Ernani de São Thiago, Universidade Federal de Santa Catarina, Florianópolis, SC, Brazil; 3 PhD, Adjunct Professor, Departamento de Enfermagem, Universidade Federal de Santa Catarina, Florianópolis, SC, Brazil; 4 RN, Hospital Universitário Polydoro Ernani de São Thiago, Universidade Federal de Santa Catarina, Florianópolis, SC, Brazil; 5 Physician, Hospital Universitário Polydoro Ernani de São Thiago, Universidade Federal de Santa Catarina, Florianópolis, SC, Brazil

**Keywords:** Patient Safety, Organizational Culture, Neonatal Nursing, Intensive Care Units, Neonatal

## Abstract

**OBJECTIVE:**

to verify the assessment of the patient safety culture according to the function and length of experience of the nursing and medical teams at Neonatal Intensive Care Units.

**METHOD:**

quantitative survey undertaken at four Neonatal Intensive Care Units in Florianópolis, Brazil. The sample totaled 141 subjects. The data were collected between February and April 2013 through the application of the Hospital Survey on Patient Safety Culture. For analysis, the Kruskal-Wallis and Chi-Square tests and Cronbach's Alpha coefficient were used. Approval for the research project was obtained from the Ethics Committee, CAAE: 05274612.7.0000.0121.

**RESULTS:**

differences in the number of positive answers to the Hospital Survey on Patient Safety Culture, the safety grade and the number of reported events were found according to the professional characteristics. A significant association was found between a shorter Length of work at the hospital and Length of work at the unit and a larger number of positive answers; longer length of experience in the profession represented higher grades and less reported events. The physicians and nursing technicians assessed the patient safety culture more positively. Cronbach's alpha demonstrated the reliability of the instrument.

**CONCLUSION:**

the differences found reveal a possible relation between the assessment of the safety culture and the subjects' professional characteristics at the Neonatal Intensive Care Units.

## Introduction

Patient safety is related to factors like human fallibility, weaknesses in health organizations, problems with technological devices, communication and inappropriate dimensioning between teams and professionals, as well as with the task burden and limited knowledge about safety^(^ 1 ^)^.

One of the safety pillars is the institutional culture, which is based on good communication, trust, organizational learning, commitment of the hospital management to safety, leadership, non-punitive approach of errors and a shared perception of the importance of this theme^(^ 2 ^-^ 3 ^)^. Therefore, the health institutions need to promote a culture based on these values to improve the patient safety.

Recently, a proposal for cultural changes related to safety stood out in Brazil through Article 5 of Ministry of Health Decree 529, issued on April 1^st^ 2013, which defines the strategies for the implementation of the National Patient Safety Program. These strategies include the promotion of the safety culture, which emphasizes learning and organizational improvement, the engagement of professionals and patients in accident prevention, with emphasis on safe systems, thus avoiding individual accountability processes^(^ 4 ^)^.

The literature shows that the assessment of the patient safety culture differs among the hospital organizations depending on the size, accreditation, specialty, and can vary among the professionals, depending on the function, length of experience and age^(^ 5 ^-^ 9 ^)^.

In order to promote a culture that sets patient safety as a priority, however, first, it is fundamental to assess the patient safety culture present in the health organizations, in order to plan specific intervention in search of better results^(^ 3 ^)^.

Identifying the factors that further the patient safety culture and the barriers that interfere in it permits the development of strategies according to the particularities of each place of work, obtaining more satisfactory results^(^ 10 ^-^ 11 ^)^.

Therefore, as the discussion of the patient safety culture is recent in Brazil, the scientific knowledge on this theme is incipient, mainly in specialized services like the Neonatal Intensive Care Units (NICU). Hence, studies are needed that focus on the safety culture at Brazilian NICUs, as these environments can entail greater risks for patient safety, due to the infants' particularities, intense care, technological devices and the professionals' knowledge and specific skills. Thus, strategies can be proposed and distinguished care technologies can be used at these units.

In that sense, the objective in this research was to verify the assessment of the patient safety culture according to the function and length of experience of the nursing and medical teams at Neonatal Intensive Care Units.

## Method

A descriptive and exploratory, quantitative, cross-sectional survey was undertaken at four NICU from four Public Hospitals, located in Florianópolis, Santa Catarina, Brazil. The population consisted of the nursing and medical team at the four NICU, totaling 181 professionals. An intentional, non-probabilistic sample was used, in line with the following inclusion criteria: being a nurse, nursing technician, auxiliary nurse or physician; working at the NICU during the data collection period; professionals hired for the NICU more than two months earlier, considering this period as the minimum length to adapt to the sector; returning the data collection instrument. The following exclusion criteria were considered: professionals from other categories than the nursing or medical team, as the sampling for the other professionals working at the NICU was not representative; professionals on holiday, health leave, pregnancy leave, sabbatical and/or medical certificate; more than half of the instruments left blank; and/or abandonment of consent.

In line with these criteria, 151 individuals were invited to participate in the study. Among this initial total number, 142 returned the data collection instrument. As one of the questionnaires had more than half of its contents left blank, the final sample consisted of 141 participants, representing 93.4% of the initial total number invited.

The data were collected between February and April 2013 through the application of the Agency for Healthcare Research and Quality's (AHRQ) Hospital Survey on Patient Safety Culture (HSOPSC), which consists of 42 items related to the patient safety culture, besides requesting the subjects' opinion on the patient safety grade at their service, the number of events reported and the respondents' professional characteristics^(^ 3 ^)^.

The AHRQ authorized the translation of application of this instrument. For the translation, the guidelines from the document recommended by the questionnaire authors were used, Translation Guidelines for the AHRQ Surveys on Patient Safety Culture^(^ 12 ^)^. Although this study was not aimed at validating the instrument, in order to avoid any noise due to the translation, the coherence of the produced translation was verified by consulting the translation to Portuguese produced by other authors^(^ 13 ^-^ 14 ^)^, showing that the questions had the same meanings.

The data collection started when the research project was presented to the professional. Next, they received the Informed Consent Form (ICF) and an envelope with the data collection instrument, which the participants completed and placed in a box.

For the descriptive analyses, the answers were recoded as positive: I completely agree, I agree, always and frequently; negative: I completely disagree, I disagree, never and rarely; neutral: I neither agree nor disagree^(^ 3 ^)^. For inferential statistical analysis, the software R 3.0.1^(^ 15 ^)^ was used, considering a significance level of p≤0.05. The tests used were Kruskal-Wallis (KW), followed by the Wilcoxon (W) test with Bonferroni's correction; and the Chi-square test. Also, as it is a North American questionnaire that was translated to Portuguese, it was considered relevant to test its reliability for reproduction in new Brazilian studies. Therefore, Cronbach's Alpha test was used, which permits analyzing the reliability of the internal consistency of the questionnaire, defined as how consistently the instrument measures a certain characteristic, ranging between 0.00 and 1.00, with a minimum of 0.60^(^ 16 ^-^ 18 ^)^.

The ethical aspects were addressed in compliance with the standards and regulatory guidelines defined in National Health Council Resolution 466/2012 - Ministry of Health. The research project was submitted to Plataforma Brasil and approval was obtained from the Ethics Committee, CAAE: 05274612.7.0000.0121.

## Results

In this study, the statistical difference was verified in the number of positive answers obtained in the HSOPSC, safety grade and number of reported events according to the subjects' professional characteristics, which included: *Length of experience at the hospital; Length of experience at the unit; Length of experience in the profession; *and* Function*. It was observed that, among the 141 subjects, 58(41%) were nursing technicians. As regards the *Length of experience at the hospital*, 71(50%) professionals had less than ten years of experience and, concerning the *Length of experience at the unit*, 76(54%) had worked at the NICU for up to 10 years. Most of the subjects, that is, 93(66%) had more than 10 years of experience in the profession (Table 1).

**Table 1:** - Function and length of experience of the nursing and medical teams at Neonatal Intensive Care Units, Florianópolis, SC, Brazil, 2013

Professional characteristics of the subjects	Characteristics [n (%)]					
	Length of experience (years)					
	<1	1-5	6-10	11-15	16-20	≥21
At the Hospital	16 (11)	29 (21)	26 (18)	18 (13)	27 (19)	24 (17)
At the Unit	21 (15)	31 (22)	24 (17)	21 (15)	24 (17)	20 (14)
In the Profession	3 (2)	26 (18)	18 (13)	33 (23)	21 (15)	39 (28)
	Functions					
	Nurse	Nur. Tech.	Aux. Nur.	Phys.	General total	
	23 (16)	58 (41)	12 (9)	48 (34)	141 (100)	

To verify the difference between the positive answers obtained on the HSOPSC in relation to the length of experience and the function, the total number of positive answers obtained on the 141 instruments was analyzed. Thus, considering the 42 instrument items, in total, 2546 (43%) positive answers were obtained.

When analyzing the results to reflect a possible relation between them, it was observed that the characteristic *Length of experience at the hospital* showed a significant difference in relation to the number of positive answers. According to Table 2, it was observed that, when the subjects had less than one year of experience at the hospital, the percentage of positive answers tended to be higher (59%) than for participants with more than one year of experience, who obtained an average 40% of positive answers.

**Table 2:** - Positive answers according to function and length of experience of nursing and medical teams at Neonatal Intensive Care Units, Florianópolis, SC, Brazil, 2013

Length of experience/Functions			n (%)	*A priori* (KW)			*A posteriori* (W)	
				x²	p		dif.	p
Length of experience at the hospital	[a]	< 1 year	401 (59)	46.19	<0.01		[a]; [b]	<0.05
	[b]	1-5 years	487 (40)				[a]; [c]	<0.05
	[c]	6-10 years	462 (43)				[a]; [d]	<0.05
	[d]	11-15 years	307 (40)				[a]; [e]	<0.05
	[e]	16-20 years	520 (47)				[a]; [f]	<0.05
	[f]	≥ 21 years	389 (40)					
Length of experience at the unit	[a]	< 1 year	460 (52)	23.53	<0.01		[a]; [b]	<0.05
	[b]	1-5 years	554 (43)				[a]; [f]	<0.05
	[c]	6-10 years	427 (43)				[e]; [f]	<0.05
	[d]	11-15 years	385 (44)					
	[e]	16-20 years	450 (45)					
	[f]	≥ 21 years	304 (38)					
Length of experience in the profession	[a]	< 1 year	56 (44)	8.86	0.12			
	[b]	1-5 years	488 (45)					
	[c]	6-10 years	382 (50)					
	[d]	11-15 years	577 (42)					
	[e]	16-20 years	374 (43)					
	[f]	≥ 21 years	689 (43)					
Functions	[a]	Nurse	373 (38)	15.55	<0.01		[a]; [d]	<0.01
	[b]	Nursing Technician	1059 (42)				[c]; [d]	<0.05
	[c]	Auxiliary Nurse	189 (38)					
	[d]	Physician	959 (46)					

Also according to Table 2, when analyzing the characteristic *Length of experience at the unit,* it was observed that the subjects with less than one year of experience obtained a higher percentage of positive answers (52%) when compared to participants with between 1 and 5 years of experience (43%).

When the condition *Length of experience in the Profession* was analyzed, no significant difference was found in the number of positive answers.

The analysis of the different functions showed that the function *Physician *presented the highest percentage of positive answers (46%) and, thus, differed statistically from the percentage of positive answers found for the functions *Nurse *and *Auxiliary Nurse*, each with 38% of positive answers.

In addition, the results also showed that some characteristics of the subjects influenced the choice of the grade, except for *Length of experience at the hospital* and *Length of experience at the unit*. Among these characteristics, *Length of experience in the profession *stands out, revealing a significant difference for the grades *Very good *and *Regular*. According to Table 3, the safety grade *Very Good *was more frequent among subjects with more than 25 years of experience in the profession (31%). The grade *Regular*, on the other hand, was more frequent among subjects with more than 11 years of experience, totaling 44 (69%).

**Table 3:** - Patient safety grades according to function and length of experience of the nursing and medical teams at Neonatal Intensive Care Units, Florianópolis, SC, Brazil, 2013

Length of experience/Functions		Patient safety grades [n (%)] and statistical significance *				
		Excellent	Very good	Regular	Bad	Very bad
Length of experience at the hospital						
	<1	3 (75)	8 (15)	3 (6)	1 (6)	0 (0)
	1-5	0 (0)	7 (13)	8 (15)	4 (25)	0 (0)
	6-10	0 (0)	12 (22)	10 (19)	4 (25)	0 (0)
	11-15	0 (0)	4 (7)	12 (21)	3 (19)	0 (0)
	16-20	0 (0)	12 (22)	11 (23)	2 (13)	1 (50)
	≥ 21	1 (25)	11 (20)	9 (17)	2 (13)	1 (50)
Length of experience at the unit						
	<1	3(100)	8 (15)	7 (10)	2 (13)	0 (0)
	1-5	0 (0)	12 (22)	16 (25)	3 (19)	0 (0)
	6-10	0 (0)	10 (19)	10 (16)	4 (25)	0 (0)
	11-15	0 (0)	5 (9)	12 (19)	4 (25)	0 (0)
	16-20	0 (0)	12 (22)	9 (14)	1 (6)	2 (100)
	≥ 21	0 (0)	7 (13)	10 (16)	2 (13)	0 (0)
Length of experience in the profession			0,01	0,01		
	<1	0 (0)	1 (2)	2 (3)	0 (0)	0 (0)
	1-5	1 (25)	11 (20)	9 (14)	4 (27)	1 (50)
	6-10	1 (25)	6 (11)	9 (14)	0 (0)	1 (50)
	11-15	1 (25)	11 (20)	16 (25)	5 (33)	0 (0)
	16-20	0 (0)	8 (15)	10 (16)	3 (20)	0 (0)
	≥ 21	1 (25)	17 (31)	18 (28)	3 (20)	0 (0)
Functions			0,01	0,01		
	Nurse	0 (0)	7 (13)	10 (16)	5 (31)	1 (50)
	Nursing Technician	3 (75)	24 (44)	24 (38)	5 (31)	1 (50)
	Auxiliary Nurse	0 (0)	4 (7)	7 (11)	1 (6)	0 (0)
	Physician	1 (25)	19 (35)	23 (36)	5 (31)	0 (0)

* Chi-square test; only significant coefficients were demonstrated and should be interpreted per column.

The analysis of the *Functions *in relation to the possible safety grades revealed a statistical difference between *Very good *and *Regular*. In that sense, the *Nursing technician *and the *Physician *were the functions that most frequently chose *Very good* (24% and 19%, respectively). The functions *Nurse *and *Auxiliary nurse *were the functions that least considered these two patient safety grade options.

In view of the number of events reported in the last 12 months, a difference was found according to the professional characteristics, except for *Length of experience at the hospital*. According to Table 4, when analyzing the *Length of experience at the unit,* a significant difference was found for the option between 1 and 2 events in relation to the others, in which the participants that most indicated that option had between 1 and 5 years of experience at the unit (33%).

**Table 4:** - Reported events according to length of experience and function of nursing and medical teams at Neonatal Intensive Care Units, Florianópolis, SC, Brazil, 2013

Length of experience/Functions		Number of reported events [n (%)] and statistical significance *					
		None	1 to 2	3 to 5	6 to 10	11 to 20	≥21
Length of experience at the hospital		-	-	-	-	-	-
	<1	5 (11)	8 (15)	3 (6)	1 (6)	0 (0)	0 (0)
	1-5	12 (27)	7 (13)	8 (15)	4 (25)	0 (0)	0 (0)
	6-10	8 (18)	12 (22)	10 (19)	4 (25)	0 (0)	0 (0)
	11-15	7 (16)	4 (7)	12 (21)	3 (19)	0 (0)	1 (25)
	16-20	10 (22)	12 (22)	11 (23)	2 (13)	1 (50)	2 (50)
	≥ 21	3 (7)	11 (20)	9 (17)	2 (13)	1 (50)	1 (25)
Length of experience at the unit		-	0,04	-	-	-	-
	<1	8 (17)	7 (16)	2 (7)	1 (14)	0 (0)	0 (0)
	1-5	8 (17)	15 (33)	7 (24)	0 (0)	1 (20)	0 (0)
	6-10	9 (20)	5 (11)	6 (21)	3 (43)	1 (20)	0 (0)
	11-15	6 (13)	9 (20)	3 (10)	1 (14)	1 (20)	1 (25)
	16-20	7 (15)	6 (13)	5 (17)	2 (29)	1 (20)	3 (75)
	≥ 21	8 (17)	3 (7)	6 (21)	0 (0)	1 (20)	0 (0)
Length of experience in the profession		0,01	0,04	0,02	-	-	-
	<1	1 (2)	1 (2)	0 (0)	0 (0)	0 (0)	0 (0)
	1-5	6 (13)	13 (29)	5 (18)	2 (29)	0 (0)	0 (0)
	6-10	7 (15)	5 (11)	1 (4)	1 (14)	1 (17)	1 (25)
	11-15	12 (26)	10 (22)	7 (25)	1 (14)	2 (33)	1 (25)
	16-20	4 (9)	8 (18)	6 (21)	2 (29)	2 (33)	0 (0)
	≥ 21	16 (35)	8 (18)	9 (32)	1 (14)	1 (17)	2 (50)
Functions		0,01	0,01	-	-	0,03	-
	Nurse	6 (13)	5 (11)	7 (24)	3 (43)	1 (20)	1 (25)
	Nursing Technician	22 (48)	22 (49)	7 (24)	3 (43)	0 (0)	1 (25)
	Auxiliary Nurse	5 (11)	1 (2)	3 (10)	0 (0)	0 (0)	1 (25)
	Physician	13 (28)	17 (38)	12 (42)	1 (14)	4 (80)	1 (25)

* Chi-square test; only significant coefficients were demonstrated and should be interpreted per column.

Concerning the characteristic *Length of experience in the profession*, the subjects that most frequently chose *No *event possessed more than 21 years of experience in the profession (35%). With respect to the *Functions, *the *Nursing Technicians *most frequently chose *None* (48%) and between *1 *and* 2* (49%) when compared to the other functions. In addition, a significant difference was found for the option *between 11 and 20 events*, in which the functions *Physician *and *Nurse *most frequently chose this number of events (80% and 20%, respectively). The other functions did not declare that option.

Cronbach's Alpha demonstrated a range from 0.43 to 0.88 among the twelve items in the instrument, with *General perceived patient safety, Staff *and* Non-punitive response to error *scoring 0.43, 0.46 and 0.47, respectively, indicating a moderate to low reliability of the internal consistency. The other nine dimensions were considered moderate to strong, with Alpha coefficients superior to 0.60 (Table 5).

**Table 5:** - Result of Cronbach's Alpha test of twelve patient safety culture dimensions of the data collection instrument - Hospital Survey on Patient Safety Culture

Dimension	Alpha
1. Teamwork at the unit	0.61
2. Expec. and actions of supervisor/head to promote patient safety	0.74
3. Organizational learning-continuous improvement	0.74
4. Hospital management support to patient safety	0.60
5. General perceived patient safety	0.43
6. Feedback and reporting about errors	0.72
7. Openness to reporting	0.64
8. Frequency of reported events	0.88
9. Teamwork among hospital services	0.60
10. Staff	0.46
11. Internal transfers and shift change	0.64
12. Non-punitive response to error	0.47

## Discussion

The patient safety culture at health services is represented by the set of knowledge, thoughts, beliefs, habits, customs and routines followed and shared among the team members^(^ 19 ^)^. Thus, many individuals can develop good or bad habits according to the safety climate in the environment they are inserted in^(^ 2 ^)^. In view of this aspect, it was considered that the length of professional experience, as well as the length of experience at the institution or unit, can provide distinct perceptions of the safety culture.

Hence, in view of the results, it was observed that, as the professionals gained experience, at the institution as well as the NICU, they chose less positive answers with regard to the safety culture. In contrast, a study involving 136 professionals assessed the relation between the answers obtained on the HSOPSC and the professionals' age, detecting that the youngest nurses (between 23 and 43 years) chose less positive answers in terms of safety when compared to the oldest ones (between 44 and 65 years)^(^ 6 ^)^.

Another professional characteristic that influences the number of positive answers was the professional function, where the physicians and nursing technicians showed a higher percentage of positive answers than nurses and auxiliary nurses at the NICU. In that sense, a study of 1113 professionals from Spanish hospitals also showed that the function was a factor associated with the assessment of the safety culture, but the nurses obtained a more positive perception^(^ 20 ^)^. In addition, according to another study undertaken in different countries, while the surgeon believed that teamwork in the surgery room was strong and, hence, assessed the culture more positively, the other members disagreed^(^ 21 ^)^.

In addition, individuals from the same professional category may perceive the safety culture differently. According to a study of 550 nurses from the United States and Canada, despite belonging to the same profession, the professional position inside the organization influenced the perceived safety. The nurses in management or administrative functions assessed the safety culture more positively than the clinical nurses^(^ 8 ^)^.

As regards the patient safety grade, a difference was found in relation to the Length of experience in the profession, with professionals with greater experience showing a greater trend to consider the safety as regular or very good. No difference was found, however, when considering the Length of experience at the hospital in relation to the grade chosen. In that sense, a study of 1460 professionals from public hospitals in Palestine, using the HSOPSC, assessed that the length of experience at the hospital did not show any significant difference in relation to the grade either^(^ 22 ^)^.

The choices of the safety grade also differed according to the function, in which the nursing technicians and physicians chose *Very Good *and *Regular *more frequently when compared to the other categories. In a study at Spanish hospitals, on the other hand, the nurses were more positive with regard to the grade than the physicians^(^ 20 ^)^.

Concerning the reporting of events, it was observed that the professionals who chose a smaller number of reported events had longer experience. As regards the functions, the technicians and physicians were the categories that most chose *None *or *1 *to* 2 *events. This result was also identified in the study undertaken in Lebanon, as mentioned earlier, where the nursing technicians were the category that most reported *No* event^(^ 9 ^)^.

As observed, the reporting of events is not very frequent at the NICU, which ends up not reflecting the actual number of errors, making barriers against these errors hardly effective. Although the National Patient Safety Program was being launched at the time of the data collection, as a national policy that more strongly emphasized event reporting did not exist until then, it should be highlighted that the hospitals that participated in the study did not present institutional strategies for these reports.

A study shows that the reporting of events faces different barriers, including the alienation of professionals out of fear of the process the reporting triggers, as well as the lack of understanding of how this report should be done, and the lack of feedback received on the information transmitted, causing lack of motivation^(^ 23 ^)^.

The low compliance with error reporting may also be related to the conducts aimed at the professionals, mainly to the punitive error approach, as shown in a study of 70 Intensive Care nurses, where 52(74%) reported that punishment happens in most cases when an error is reported^(^ 24 ^)^.

Besides these results, as the data were collected before the validation of the HSOPSC for the Portuguese language^(^ 14 ^)^, the reliability of the internal consistency of our translation was verified. First, it should be highlighted that the authors of the HSOPSC developed a pilot test to apply this instrument at 21 American hospitals, involving 1437 health professionals, with Alpha coefficients ranging between 0.63 and 0.84^(^ 3 ^)^.

The results of the analyses in our study showed that, in the dimensions *General perceived patient safety*, *Staff* and *Non-punitive response to error, *the Cronbach's Alpha scores were inferior to 0.60, indicating low internal consistency in these dimensions. In a study that involved 322 professionals, the Alpha for these last two dimensions corresponded to 0.20 and 0.35^(^ 25 ^)^, while the present results equaled 0.46 and 0.47, which are nevertheless considered low. In contrast, the dimension *General perceived patient safety *showed an Alpha coefficient of 0.52^(^ 25 ^)^, while the present result was equal to 0.43.

Low Alpha coefficients were also found in the validation of the Turkish (n=309), Spanish (n=174), Dutch (n=583) and Japanese (n=6395)^(^ 25 ^)^ versions of the instrument though. These studies highlight that these results may be influential due to the sample size as, the larger the sample, the greater the chances of repetition in the analysis of the Alpha will be and, finally, the higher the Alpha coefficient may be^(^ 18 ^)^. Therefore, the HSOPSC should be used in other studies in Brazil, as it is only by using the instrument in different samples that its validity and reliability can be confirmed^(^ 25 ^)^.

## Conclusion

The patient safety cultures as NICUs is considered associated with individual as well as collective factors, that is, the way of thinking, acting and doing safety at the workplace. Hence, the formation of this cultural perspective is collectively constructed over time and in the course of the experiences in the workplace, where each professional contributes with his/her values and shares the priority of patient safety.

The safety culture can also receive influence from the professionals' function and Length of experience in the profession, as well as from the length of experience at a certain institution or unit, starting to make their decisions according to the predominant safety climate at each location.

The recent specific public policies on the theme reveal the start of the construction of new cultural perceptions about the patient safety. Prioritizing these policies, the health system should provide for patient safety strategies, one of which is error reporting. Through error reporting and the non-punitive culture, the causal factors can be identified and barriers can be implemented that reduce the risk situations.

The size of the population and sample to assess the reliability of the instrument was considered a limitation in this study. Nevertheless, it is considered that the HSOPSC permits analyzing the safety culture at institutions and identifying its characteristics. Therefore, this instrument can be used in the Brazilian health context, through the replication of research in NICUs, with a view to exploring the patient safety culture in these environments in further depth, as well as through its application in other hospital populations.
